# Household food insecurity and its association with nutritional status of under five children in Sekela District, Western Ethiopia: a comparative cross-sectional study

**DOI:** 10.1186/s40795-017-0149-z

**Published:** 2017-04-08

**Authors:** Ermiyas Mulu, Bezatu Mengistie

**Affiliations:** 1 Department of Public Health, College of Medicine and Health Sciences, Ambo University, P.O. Box, 19, Ambo, Ethiopia; 2 Bezatu Mengistie, College of Health and Medical Sciences Haramaya, Haramaya University, Harar, Ethiopia

**Keywords:** Food insecurity, Under five children, Stunting, Underweight, Wasting, Ethiopia

## Abstract

**Background:**

Food insecurity influences children nutritional status by limiting the quantity and quality of dietary intake. Studies conducted across different parts of the world revealed controversial evidences about the relationship between household food insecurity and child nutritional status. Although child malnutrition and food insecurity are the main problems in Ethiopia, to what extent food insecurity contributes to children nutritional status is not yet well studied. Therefore, this study was conducted to compare children nutritional status in food secure and insecure housholds.

**Method:**

A community based comparative cross sectional study was conducted in Sekela District,Western Ethiopia from February 5–27, 2014. The total sample size was 576 households having at least one children less than 5 year’s old. Two stage cluster stratified sampling technique was used to select study participants. Data were collected using a pre tested structured questionnaire and anthropometric measurements. Household food insecurity was measured using household food insecurity access scale. Anthropometry indices were calculated using WHO Anthro 3.1.0 and interpreted according to WHO 2006 cutoff points. Data were entered using Epi.Data 3.2. and exported to SPSS 21.0 for analysis. Logistic regression analysis was employed to identify independent predictors of children under nutrition.

**Result:**

The mean of household food insecurity score was 8.16 ± 6.01 and the prevalence of food insecurity was 74.1%. Of children in food insecure households 38.9% were stunted, 22.6% were underweighted and 12.9% were wasted while the respective prevalence of stunting, underweight and wasting were 31.3%, 11.8% and 7.6% among children in food secure households. Food insecurity had association with children underweight (AOR = 2.25; 95% CI = 1.29, 3.94), but not with stunting and wasting. Children under nutrition had independent association with sex and age of the child, colostrum feeding, upper respiratory infection, fever, and maternal literacy.

**Conclusion:**

Household food insecurity and child under nutrition were critical problems in the study setting. Socio demographic factors, poor child caring practices, infection and food insecurity had positive association with children under nutrition. Thus, due emphasis should be given for the designing and implementation of multi sectorial community based nutrition interventions and initiation of income generating livelihood to the community to curtail under nutrition and household food insecurity in the locality.

## Background

Child under nutrition is alarming public health problem in developing countries [1]. Several and multifaceted factors influence nutritional status of children in developing countries. According to United Nations International Children’s Emergency Fund (UNICEF) conceptual framework of causes of children malnutrition, household food insecurity, inadequate child care and unhealthy household environment and lack of health services are the underlying cause of children under nutrition [2].

Ethiopia achieved successive economic growth and expansion of health services in the past one decade. However, under nutrition remains a highly prevalent public health problem among children of under 5 years age. Underlying poverty and rapid population growth exacerbate the gap between basic health services, food and nutrition [3, 4]. The national data on the nutritional status of under five children indicated that high prevalence of undernutrition, 40% stunting, 27% underweight and 9.7% wasting [5].

Diet quantity, diet quality, food poverty and food shock indicators generated from food security and vulnerability analysis of Ethiopian Central Statistical Agency and World Food programme in 2014 revealed that food insecurity is widespread and severe problem in Ethiopia [6]. Food insecurity influences nutritional and health status of household members by limiting the quantity and quality of dietary intake [2]. Studies in developed and developing countries revealed inconsistent evidence about the influence of household food insecurity on children nutritional status [7–12]. High prevalence of children malnutrition was reported in surplus crop producing areas of western Ethiopia, factors other than food security contribute to under nutrition [13]. Although food insecurity and children under nutrition are common problems in Western Ethiopia, to what extent food insecurity contributes to children nutritional status is not well studied [13–15]. Hence, this study aimed to compare nutritional status of children in food secure and food insecure households in Sekela District, Western Ethiopia.

## Methods

The study was conducted in Sekela District, Western Ethiopia. It is located about 440 km northwest of the capital Addis Ababa. Agriculture, mainly cereal crop production, is the means of livelihood for the majority of inhabitants. The main types of crops produced are teff (*Eragrostis tef*), bean, barely, oat and cowpea [16]. The district has four distinct seasons; spring (September- November); winter (December – February), autumn (March- May) and summer (June-August). The end of spring and the beginning of winter is the harvesting time. The data collection period was during the post-harvest season.

A community based comparative cross sectional study was conducted among under five children living in food secure and food insecure households from February 5–21, 2014 in Sekela District, Western Ethiopia. Sample size was determined considering P_1_ = 0.487 and P_2_ = 0.32 [13], confidence level of 95%, power of 80% and using the following formula.

$$ \frac{{\mathrm{n}}_1={\mathrm{n}}_2={\left\{{\mathrm{Z}}_{\upalpha /2}\sqrt{2\mathrm{P}\left(1-\mathrm{P}\right)}\kern0.5em +\kern0.5em {\mathrm{Z}}_{\upbeta}\sqrt{\mathrm{P}1\left(1-\mathrm{P}1\right)\kern0.5em +\kern0.5em \mathrm{P}2\left(1-\mathrm{P}2\right.}\right\}}^2}{{\left(\mathrm{P}1-\mathrm{P}2\right)}^2} $$

Where; P_1_ and P_2_ denotes proportions of event of interest (outcome) for group I and group II, P denotes *P*_*1*_ *+ P*_*2*_/2, Z_α/2_ denotes normal deviate at a level of significance and Z_1-β_ denotes the normal deviate at 1-β% power with β% of type II error.

Based on the above formula, assumptions and parameters, the calculated sample size was 262. We considered a design effect of two and a nonresponse rate of 10% and the total sample was 576 households with under five children. Study participants were selected using two stage stratified cluster sampling technique. Villages in Sekela District were stratified by agro-ecological zones. There are 27 villages in the district; of which 21villages are cool humid mid highlands & six villages are cool sub-humid mid highland [17]. Using a lottery method, one village was selected from six cool sub-humid mid highland villages and three villages were selected from 21 cool humid mid highland villages. The lists of households with under five children in the four villages were obtained by census two weeks prior to the actual data collection. From the organized list, 576 households with at least one under five children were selected using simple random sampling technique. If more than one under five children were living in a household, one was selected using a lottery method. Children with diagnosed chronic illness/bed ridden, caught serious acute disease, and had physical deformity in lower extremity and spine were excluded from the study.

Structured and pretested questionnaire was used to collect data on socio demographic characteristics, environmental condition, child feeding habit, illness in past two weeks and the use of preventive health care services. Age of the child was taken from growth monitoring and immunization card. For those who missed the card, their age were recorded relying on the date given by the mothers or caretakers. The questionnaire was initially prepared in English and translated to Amharic (local language) version by fluent speakers and back to English to check the consistency. The data collectors and supervisors were given two days intensive training about obtaining informed consent and participants’ rights, interview techniques, anthropometry measurements and use of survey instruments. Supervisors had checked the data collection process and filled questionnaires daily to ensure accuracy of the data.

Household food insecurity was measured using household food insecurity access scale (HFIAS) developed mainly by Food and Nutrition Technical Assistance (FANTA) for use in developing country settings. The tool consisted of nine questions that represent generally increasing level of severity of food insecurity and four frequency of occurrence. The nine generic occurrence question relate to three domains of food insecurity. The first generic question relates to anxiety and uncertainty about the household food supply; the next three generic question relate to insufficient diet quality; and the rest five generic question relate to insufficient food intake and its physical consequences [18]. We used the Amharic version of HFIAS questionnaire, which was previously adapted and validated in Ethiopia [19]. Before data collection begun, the Amharic version of the questionnaire was pretested on 58 households in the nearby village of the study area. There was also a focused group discussion with eight key informants who were familiar with the conditions and experiences of household food insecurity in the area. Some type of food items in the HFIAS questions, that provide locally relevant examples when the respondent requires further prompting, were modified to suit the study setting contexts. The reliability test indicated that our HFIAS tool had an adequate internal consistency (Cronbach’s alpha = 0.88).

Anthropometric measurements were taken from children. For children aged 6–23 months length was measured in a recumbent position to the nearest one millimeter using length board. For children 24 months older height was measured in a standing-up position to the nearest one millimeter using height measuring board. Weight of child was measured to the nearest 10g for children aged 6–23 months using salter scale and to the nearest 100 g using electronic weighing scales for a child 24 months and older. Data collectors had taken at least two height and weight measurements for an individual. They had repeated the measurements when the variation of the two measures was greater than 0.1 kg for weight and greater than a 0.1 cm for height. Functionality of digital scales were checked using known weight every morning before data collection begun. Data collectors assured the scale reading exactly at Zero before every weight measurement. Data collectors’ accuracy of anthropometric measurements were standardized to the desired precision with their trainer/through repeated measurements during training and pretesting.

The data were checked for completeness, cleaned and double entered into Epi Data 3.1and exported to Statistical Package for Social Science 21.0 for further analysis. Based on responses given to the nine questions and frequency of occurrence over the past 30 days, households were assigned a score that ranges from 0 to27. A higher HFIAS score is indicative of poorer access to food and greater household food insecurity. A household was classified as food secure if respondent did not experience none of the food insecurity conditions, or just experienced worry rarely, otherwise as food insecure. Food insecure households were further classified as mild, moderate and severe food insecure as follows. A household was classified as mildly food insecure if the respondent was worried about not having enough food sometimes or often, and/or any of the household’s member rarely scarified quality of dietary intake. A household was classified as moderately food insecure if any of the household’s members sacrificed quality more frequently and/or had started to cutting back on quantity by reducing the size of meals or number of meals, rarely or sometimes. A household was classified as severely food insecure if any of the household’s member was graduated to cutting back on meal size or number of meals often, and/or experiences any of the three most severe conditions (running out of food, going to bed hungry, or going a whole day and night without eating), even as infrequently as rarely [18]. Respondents’ response to the nine HFIAS questions and their frequency of occurrence were summarized using frequency table, and mean ± SD (standard deviation) and median HFIAS scores. Children’s characteristics were compared by household food insecurity using *t*-test and Chi-Square test.

Nutrition indices were computed using WHO Anthro 3.1.0 and the results were classified according to World Health Organization 2006 cut-off points [20]. The Length/height for age Z-score (HAZ), weight for age Z-score (WAZ) and weight for length/height Z-score (WHZ) of children were calculated. The mean HAZ, WAZ and WHZ of children living in food secure and insecure households were compared using *t*-test. The outcome variables, nutritional status of under five children, were defined as follows by using WHO growth standards. Children whom HAZ-score less than -2SD (standard deviation) were stunted, WAZ-score less than -2SD were underweight and WHZ less than -2SD were wasted. Those children with HAZ, WAZ and WHZ scores greater than or equal to -2SD were considered as normal.

The multicollinearity of independent variables were assessed using variable inflation factor (VIF). Binary logistic regression model was used to identify the independent predictors of children under nutrition. Statistical association was asserted based on 95% CI and two sided 5% level of significance (α < 0.05). Bivariate analysis was conducted to assess the relationship between outcome variables and explanatory variables. Variables with p value < 0.2 in bivariate analysis entered in to the multivariate analysis to control all possible confounders and to detect true predictors of stunting, underweight and wasting.

A letter of ethical clearance was obtained from Haramaya University College of Health and Medical Sciences Institutional Health Research Ethical Review Committee. The purpose, risk, benefit, confidentiality, nature of the study and their degree of involvement were fully explained to parents or caregivers by the local language. Data were collected after the parent/caregiver agreed and signed the informed written consent. Severe acutely malnourished children in the study were identified and treated in government health institution.

## Result

### Households’ food insecurity

Out of the 576 sample study of households having under five children, 555 participated in the study with a response rate of 96.4%. The responses to the nine HFIAS questions and their frequency of occurrence is depicted at Table 1. Based on the responses to the nine generic occurrence questions, 86.1% of the households’ felt anxiety and uncertainty about the household food supply; 73.3% of the households’ had insufficient diet quality; and 56.4% of the households’ experienced insufficient food intake and its physical consequences domains of food insecurity. Out of the possible 0–27 HFIAS score, the minimum score was 0 and the maximum score was 25. The households’ mean ± SD HFIAS score was 8.16 ± 6.01 (95% CI = 7.68, 8.64) and the median score was 10. Three forth (74.1%; 95% CI = 70.4%, 77.7%) of the households had experienced signs of food insecurity 30 days prior to the survey that varies in severity. Out of all households 14.1%, 36.2% and 23.8% had experienced mild, moderate and severe food insecurity conditions, respectively (Table 1).

**Table 1:** Percentage of households that experienced specific food insecurity conditions in the past 4 weeks preceding the survey (*N* = 555) in Northwest Ethiopia

Frequency	Worry For household food supply	Lack preferred food	Ate repeated food item	Ate inferior food	Cut meal portion	Cut meal frequency	No food in the house	Go bed hungry	Stay all the day without food
Never	13.9	26.7	31.2	39.5	49.0	53.2	91.7	84.0	98.7
Rarely	17.1	14.1	13.5	18.7	31.2	27.2	5.8	12.1	.9
Sometimes	20.9	27.6	27.6	29.4	15.3	12.6	2.5	3.1	.4
Often	48.1	31.7	27.7	12.4	4.5	7.0		0.9	

### Household food insecurity and socio-demographic characteristics

The characteristics of children were compared by household food insecurity. The mean age of children was 31.39 ± 1.2 months and the sex ratio was one. Children in food insecure households had higher proportion of illness than children in food secure households two weeks prior to the survey. However, there were no such significant difference in morbidity between children in food secure and food insecure households. Food insecure households generally had lower socioeconomic status. The overall illiteracy rate was 78.7% among the mothers and 58.2% among the fathers. Food secure households had better literacy and possession of household assets. Farming was the means of livelihood for most of the households and majority of the households possessed farmland that varies in size. TV/Radio or both were more available in food secure households compared to food insecure household. About two third of the household had access to safe water. Food secure households had better child feeding practice and use of child and maternal health services compared with food insecure counterparts. Higher proportion of colostrum avoidance and prelacteal feeding, lower frequency of complementary feeding and perinatal care were observed among food insecure households compared with food secured households (Table 2).

**Table 2:** Characteristics of children in Northwest Ethiopia by household food insecurity

Characteristics	All	Food Secure(*n* = 144)	Food Insecure(*n* = 411)
Children			
Number of under five children	555	144	411
Age(Mo)	31.39 ± 1.2	30.46 ± 2.37	31.71 ± 1.34
Sex(%Male)	49.9	50	49.9
Morbidity			
Upper respiratory infection	28.7	25.2	30.4
Fever	18.9	17.1	20
Diarrhoea	20.7	19.5	21.1
Mother			
Head of household(% female)	12.4	6.3	14.6*
Education(% no formal education)	78.7	69.4	82
Work involvement(%yes)	69.5	61.1	72.5**
Father’s education(% no education)	58.2	47.9	61.8*
Household			
Roof of the house(%thatched)	16.6	4.9	20.7**
Number of room (% ≥ 3)	22.4	35.4	17.8
TV/Radio	28.1	36.8	26.0*
Electricity	6.1	14.2	3.2**
Farmland(%yes)	81.6	77.8	83
Home garden(%yes)	27.2	25.2	27.7
Animal possession(%yes)	81.5	93.1	76.9*
Safe water(%yes)	67.1	68.8	66.4
Toilet use	66.4	67	66.2
Use of preventive child health service			
Early initiation of breastfeeding(%yes)	31.4	35.8	29.6
Colostrum avoidance(%yes)	44.4	34	48.8**
Prelacteal feed(%yes)	35.4	24.5	40*
Breastfeeding on demand	63.2		
Introduction of complementary foods at 6 month	23	20.8	24
Frequency of complementary feeding(% ≥ 3 meal/day, *N* = 555)	38.8	49	34.4*
Fully immunized (%yes, *N* = 555)	66.3	75	63.3
Vitamin A Supplementation in the past 6 month (% yes)	72.1	72.9	71.8
Deworming in the past 6 months(% yes)	78.9	81.3	78.1
Use of preventive maternal health service			
ANC follow (% yes, *N* = 545)	49.7	59	46.5*
Place of delivery(% institution delivery N=)	10.6	15.3	11.9
Current family planning use(%yes)	55.3	59	54

*p < 0.05, **p < 0.01 the difference between food secure and insecure households

### Household food insecurity and children nutritional status

The overall mean ± SD height for age, weight for age and weight for height Z-scores of children were −1.51 ± 1.52, −1.12 ± 1.23 and −0.4 ± 1.50 respectively. The overall prevalence of stunting, underweight and wasting was 36.9%, 19.8% and 11.5% respectively. Children from food insecure households had lower mean Z- score for all index and higher prevalence of stunting, underweight and wasting. Of food insecure children 38.9% were stunted, 22.6% underweighted and 12.9% wasted compared with 31.3% stunted, 11.8% underweighted and 7.6% wasted of food secure counterparts. However, there was only a significant difference in the prevalence of underweight between the food insecure and food secure groups (*p* < 0.01) (Table 3).

**Table 3:** The Z-score and prevalence of children under nutrition stratified by household food insecurity

Indicator	All	Secure	Insecure
Mean ± SD HAZ- score	−1.51 ± 1.52	−1.31 ± 1.43	−1.58 ± 1.54
Stunted	36.9	31.3	38.9
Moderate(HAZ-score < −2SD and ≥ −3SD)	27.4	25.7	28.0
Severe(HAZ-score < −3SD)	9.5	5.6	10.9
Mean + SD WAZ- score	−1.12 ± 1.23	−0.88 ± 1.10	−1.21 ± 1.26
Underweight(%yes)	19.8	11.8	22.6**
Moderate(WAZ-score < −2SD and ≥ −3SD)	14.1	7.6	16.3
Severe(WAZ-score < −3SD)	5.8	4.2	6.3
Mean + SD WHZ- score	−0.4 ± 1.50	−0.22 ± 1.30	−0.45 ± 1.56
Wasting(%yes)	11.5	7.6	12.9
Moderate(WHZ-score < −2SD and ≥ −3SD)	8.1	6.3	8.8
Severe(WHZ-score < −3SD)	3.4	1.3	4.1

** *p* < 0.01 the difference between food secure and insecure households

The multivariate analysis result showed that household food insecurity had association with child underweight (AOR = 2.25; 95% CI = 1.29, 3.94) but not with stunting and wasting. Being stunted, underweight and wasted were significantly associated with sociodemographic and child characteristics. It was found that the sex and age of children had association with stunting. The odds of stunting was lower for female children (AOR = 0.64; 95% CI = 0.44, 92) compared to male counterparts. Increase in child’s age was associated with higher probability of stunting, the likelihood of stunting increased by 1.03 per one month increment on child’s age. Maternal literacy was negatively associated with child risk of stunting. Children born from literate mothers were 41% less likely (AOR = 0.59; 95%CI = 0.37, 0.95) to be stunted compared to children born from illiterate mothers. Appropriate child feeding practices were significantly associated with lower proportion of stunting. Colostrum feeding (AOR = 0.50; 95% CI = 0.35, 0.73) and frequency of child feeding (AOR = 0.63; 95%CI = 0.43, 0.93) more than twice within a day were negatively associated with stunting. In addition, child under nutrition was positively associated with child’s experience of infections two weeks preceding the data collection. Children who had upper respiratory infection were 34% more likely (AOR = 1.34; 95% CI = 1.17, 1.49) to be stunted compared to children who had not the symptom. The likelihood of underweight was 2.25 times (AOR = 2.25; 95%CI = 1.29, 3.94) higher for children who had flu symptoms compared with children who had not the symptoms. Children who had fever were 2.42 times (AOR = 2.42; 95%CI = 1.39, 4.24) more likely to be wasted compared to children who were free of the symptom in two weeks’ time prior to the survey (see Table 4).

**Table 4:** Logistic regression estimate of the effect of explanatory variables on child under nutrition

Variable	COR (95% CI)	AOR(95% CI)
Stunting		
Age of child in months	1.03(1.01,1. 19)**	1.03(1.02,1.05)**
Sex; 0 = male, 1 = female	.633(.45, .91)	.64(.44,.92)*
Maternal education; 0 = No, 1 = yes	.568(.362,.889)*	.59(37,.95)*
Vaccination; 0 = No, 1 = Yes	.74(.51,1.06)	
Frequency of child feeding 0 = ≤ 2 times/day, 1 = ≥3times/day	.76(.54, 1.08)	.63(.43,.93)*
Toilet use; 0 = No, 1 = Yes	.62(.43,.89)*	
Food insecurity; 0 = No, 1 = yes	1.40(.94,2.10)	
Initiation of breastfeeding; 0= >1 h, 1 = ≤ 1 h	.92(.65,1.10)	
Colostrum feeding; 0 = No, 1 = yes	.45(.31,.65)**	.50(.35,.73)**
Prelacteal; 0 = No, 1 = Yes	1.10(.86,1.34)	
Flue; 0 = No, 1 = yes	1.30(.85,1.98)	1.34(1.17,1.49)**
Diarrhea; 0 = No, 1 = yes	1.62(1.07,.2.46)*	
Underweight		
Sex 0 = female, 1 = male	1.52(.99,2.31)	
TV/Radio 0 = No, 1 = Yes	.40(.23,.70)**	
Colostrum 0 = No, 1 = Yes	.66(.43,1.00)	
Prelacteal 0 = No, 1 = Yes	1.39(.90,2.16)	
Vaccinated 0 = No, 1 = Yes	.65(.42,.99)	
Maternal education 0 = illiterate, 1 = literate	.79(.43, 1.34)	
Food insecurity 0 = No, 1 = Yes	2.19(1.25, 3.81)**	2.25(1.29,3.94)**
Time of initiation of breastfeeding 0 = <1 h, 1= > 1 h	.71(.43,1.16)	
Upper respiratory infection 0 = No, 1 = Yes	1.55(1.02,2.36)*	1.61(1.05,2.46)*
Wasting		
Age of the child	.98(.96,1.00)	
Fever 0 = No, 1 = Yes	2.44(1.41, 4.23)**	2.42(1.39,4.24)**
Father’s education 0 = No, 1 = Yes	1.93(1.14,3.27)*	1.61(0.93,3.83)
Maternal work involvement 0 = No, 1 = Yes	.52(.31,.88)*	
Animal possession	.58(.29,1.18)	
Prelacteal feeding 0 = No, 1 = Yes	1.71(1.01,2.91)*	1.74(1.00,3.03)
Food insecurity 0 = No, 1 = Yes	1.79(.91,3.53)	2.01(1.01,4.04)*

α significant at * *p* < 0.05, **α *p* < 0.01; *COR* crude odds ratio, *AOR* adjusted odds ratio, *CI* confidence interval

## Discussion

The results of this study showed that there is high prevalence of household food insecurity in Western Ethiopia. In agreement with the current study, other studies conducted in the district reported high prevalence of household food insecurity [14, 15]. This denotes that food insecurity is a serious and persistent problem in the study setting. This was not a surprising finding as nearly all of the participants were agrarian while the topography is hilly and not suitable for farming, the soil is prone for erosion and infertile, the size of farmland is small and there is no food security program in the area [16]. Our result indicated that food insecure households had lower socio economic status compared with food secure households. This finding demonstrated the UNICEF malnutrition conceptual frame work which includes limited availability and control of resources, educational status, gender inequality etc., affects household food access [2].

Under nutrition is a common problem of children in Western Ethiopia. The prevalence of children stunting and wasting in this study were comparable with mini EDHS 2014 report but lower than EDHS 2011 regional report and other pocket studies across the country. Underweight prevalence was lower than EDHS reports and other studies conducted in Ethiopia [5, 11, 13, 21–23].

Our main finding was household food insecurity was associated with only child underweight but not with stunting and wasting. Studies in Ethiopia and developing countries reported association between household food insecurity and child underweight [9, 11, 12, 24]. This finding was consistent with the UNICEF conceptual framework for causes of malnutrition in developing countries, household food insecurity negatively influences child nutritional status by limiting the quantity and quality of dietary intake [2].

There are controversial finding on the relationship between food insecurity and child stunting. Similar to the finding of this study, studies across developing countries revealed the absence of association between food insecurity and children stunting [9, 10, 25]. On the other hand, studies in Ethiopia and other developing countries found positive relationship between food insecurity and stunting [8, 12, 24–26]. Child under nutrition is a result of inadequate dietary intake, inadequate care and poor health of the child [2]. Our finding demonstrated the fact that food security is a necessary but not a sufficient condition to ensure good nutritional status.

The results of this study indicated that socio demographic and child characteristics had association with child under nutrition. The odds of stunting was higher for male children compared to female children. Similarly, EDHS 2011 and a study in West Gojjam reported higher prevalence of stunting among males [5, 13]. It needs a further study to identify what actually factor led the sex difference in stunting. Age of the children was also significantly associated with stunting, the likelihood of stunting increased as the child gets older. This finding is consistent with the studies in other parts of Ethiopia [13, 22]. In the 1st year of life, children have limited exposure to the environment and susceptibility to infection is lower. Breast milk is adequate to meet nutritional requirements in the first six months and continues being important source of energy and protein until second year of life. Besides, the quantity of complementary food needed is smaller for younger children that the family can access food easily for the infants and young children [27, 28].

Maternal education can improve child nutrition by increasing income and control of resources in the house. Educated mothers are likely to have better knowledge on child feeding and care and increase access to health and sanitary environment [29, 30]. Our result showed that maternal education had negative relationship with stunting. This finding agreed to other studies in developing countries that identified maternal education as an important enabling factor for under five children nutritional status [21, 24].

Infection can result under nutrition through 1.poor utilization of nutrients through indigestion, malabsorption, and poor biological use of nutrients; 2. Inadequate dietary intake and increase nutrient requirement of the body. In turn, malnutrition increases susceptibility to infection and bouts of infection to continue [31, 32]. In agreement with other studies, the finding of this study indicated positive relationship between infection and child under nutrition [11, 13, 32–34].

Suboptimal breastfeeding practices like colostrum deprivation and prelacteal feeding were common in the area. Colostrum deprivation positively associated with child stunting. Similar finding was reported from neighborhood districts [13]. Colostrum provides immune factors and act as first immunization for the child [35]. Thus, colostrum avoidance results easy exposure of child to infection in his/her earlier life and hence under nutrition.

The authors acknowledge the following limitations in this study. Firstly; information was gathered on the basis of mother’s/caregiver’s responses, it was likely to have recall bias. Secondly; since anthropometry measurements are prone to measurement bias, we might not rule out some misclassification of children’s nutritional status. However, there were standardization of anthropometric instruments, intensive training to data collectors and close supervision to overcome anthropometric measurement errors and interviewer bias.

## Conclusion

Food insecurity was widespread, persistent and sever problem in Sekela District, Western Ethiopia. Due concern should be given for designing and implementing innovative small business generating actions to curtail the existing food insecurity in the area. Food insecure households had lower socioeconomic status and poor use of child and maternal health services. Concerned bodies should give adequate attention to educate and empower women and implementation of health promotion and delivery services that best fit to the poor. Under nutrition is a critical problem in the study setting that threats children’s health, survival and their future development. The prevalence of sever acute malnutrition among children in food insecure households was alarmingly high that necessitate urgent action.

This study revealed controversial finding about the relationship between food insecurity and children under nutrition. Household food insecurity was associated with only underweight. Food insecurity, inadequate child caring practice and unhealthy environment negatively influenced dietary intake and health status of children that resulted in high child under nutrition in the locality. Thus, designing and implementing multi sectorial community based nutrition interventions should be given due attention to curb under nutrition in the district.
